# Aptamer-based gold nanoparticle aggregates for ultrasensitive amplification-free detection of PSMA

**DOI:** 10.1038/s41598-023-46974-4

**Published:** 2023-11-15

**Authors:** Giulia Matteoli, Stefano Luin, Luca Bellucci, Riccardo Nifosì, Fabio Beltram, Giovanni Signore

**Affiliations:** 1 Fondazione Pisana Per La Scienza ONLUS, Via Ferruccio Giovanetti 13, 56017 San Giuliano Terme, PI Italy; 2 National Enterprise for Nanoscience and Nanotechnology (NEST), Scuola Normale Superiore, Piazza San Silvestro 12, 56127 Pisa, Italy; 3 NEST, Istituto Nanoscienze-CNR, Piazza S. Silvestro 12, 56127 Pisa, Italy; 4 Present Address: Biochemistry Unit, Department of Biology, University of Pisa, via san Zeno 51, 56123 Pisa, Italy

**Keywords:** Biosensors, Nanostructures, Supramolecular chemistry

## Abstract

Early diagnosis is one of the most important factors in determining the prognosis in cancer. Sensitive detection and quantification of tumour-specific biomarkers have the potential to improve significantly our diagnostic capability. Here, we introduce a triggerable aptamer-based nanostructure based on an oligonucleotide/gold nanoparticle architecture that selectively disassembles in the presence of the biomarker of interest; its optimization is based also on in-silico determination of the aptamer nucleotides interactions with the protein of interest. We demonstrate this scheme for the case of Prostate Specific Membrane Antigen (PSMA) and PSMA derived from PSMA-positive exosomes. We tested the disassembly of the system by diameter and count rate measurements in dynamic light scattering, and by inspection of its plasmon resonance shift, upon addition of PSMA, finding appreciable differences down to the sub-picomolar range; this points towards the possibility that this approach may lead to sensors competitive with diagnostic biochemical assays that require enzymatic amplification. More generally, this scheme has the potential to be applied to a broad range of pathologies with specific identified biomarkers.

## Introduction

Cancer is a severe public health burden worldwide^1^, and despite new therapeutic approaches developed in the last decades, early detection is still the most important factor in determining the prognosis^2^. However, most cancers evolve unnoticed for years before being diagnosed, because in the initial lag phase, before the metabolic switch with ensuing increased cancerous cells activity and fast growth^3^, the amount of secreted biomarkers is extremely low*.* Unfortunately, the sensitivity level of available diagnostic assays used in clinics has reached its boundaries, which for ELISA assay (the gold standard in clinics for protein detection) usually lies, in clinically applicable setups, in the low picomolar range^4,5^, with important sensitivity variations depending on the used antibody and on batch variability^4,6^. There is therefore the need for alternative analytical assays, and in this scenario aptamers coupled to nanostructures represent ideal candidates to assemble sensitive and practical nanosensors^7^. Aptamers can be synthesized in large amount, with a high rate of reproducibility and with reduced production costs. Moreover, aptamers can be selected over several types of targets (from small molecules to whole cells), and they are thermally stable and more amenable to chemical modifications^8^. Finally, different aptamers structure-switching nucleotidic sequences have been developed for fluorescence^9^ or enzymatic^10,11^-based sensing reactions. On the other side, nanoparticle-based sensors represent flexible tools that can provide improved sensitivity level^12^. In particular, gold nanoparticles (AuNPs) have optical features that make them appealing, since upon interaction with light at the appropriate frequency, a localised surface plasmon resonance (LSPR) can be excited. LSPR depends on several parameters, including the AuNPs size, shape and, in the case of interacting NPs, their relative distance and mutual orientation^13^. The majority of AuNPs sensors based on the variation of colorimetric and optical proprieties rely on controlled nanoparticle aggregation in response to the presence of a target. Detection of specific proteins using this strategy was reported for both aptamer-^14,15^ and antibody-functionalised AuNPs^16^. We consider here instead a method based on the controlled disassembly of AuNPs-aggregates, which can be designed solely using nucleotides not only in the context of genetic analysis, but, when using aptamers, for the detection of other biomarkers as well. Aptamers secondary structure increases the complexity of nucleotidic switching architectures, compared to the sensing of a third complementary strand^17^, and cannot always be utilized for disassembly, as in the case of aptamers that bind different multiple epitopes on the target^18^. To date, only few aptamer-disassembly-based sensors have been reported for the detection of small molecules, even less for proteins, which represent a high fraction of the interesting biomarkers. Liu et al.^19^ developed a colorimetric assay based on AuNPs-aggregates that selectively disassemble when in presence of adenosine, since its recognition is based on a switchable dsDNA architectures that comprises an adenosine specific aptamer. Disassembly systems designed to detect proteins were reported by Ma et al. for the Prostate Specific Antigen^20^, but in that case the surface enhanced Raman signal (SERS) was the highest with the assembled system, and decreased upon disassembly. Huang et al.^21^ recently reported another colorimetric assay based on AuNPs-aggregates that separate in the presence of thrombin. However, different detection schemes can be envisioned as well, e.g. by measuring the sizes of the disaggregated clusters (e.g. by DLS) or by separating the disassembled single metal reporters from the bigger clusters; the availability of several highly sensitive analytical techniques (e.g. inductively coupled plasma mass spectrometry, ICP-MS) able to detect metal ions with sub-ppt sensitivity, could then allow ultrasensitive detection of biomarkers^22^, and the work reported here represents a first step towards the development of novel methods derived from this concept.

Disaggregating DNA-based architectures have the potential to be modified for the detection of different biomarkers, among which we are considering especially proteins; contextually, the recent development of in silico aptamer-protein interaction prediction tools provided a way to estimate binding energies and to visualise which loops of the aptamer are in contact with the protein^23^. This can be of aid in the rational development of new aptamers, in the design of 3D DNA-based structures and, most importantly in our case, in the design or optimization of the nucleotide sequences used in the complete architecture that should disassemble in the presence of the target protein.

In this work, we introduce, as proof-of-concept, a nanoparticle-based nanostructure composed of aggregated ssDNA-functionalised AuNPs that is sensitive to the presence of Prostate Specific Membrane Antigen (PSMA), a transmembrane protein here considered as a case study in view of its implications in tumour diagnosis^24–27^. The present nanostructure includes a known aptameric sequence specific to PSMA^28^ and is designed, starting from an in silico ssDNA:protein docking, to provide stable NPs aggregates that selectively disassemble in the presence of PSMA, providing a signal that we measured by Dynamic Light Scattering (DLS) and/or plasmon resonance spectroscopy. This construct has the potential to be the basis for a sensitive and time-effective nanosensor that exploits the flexibility provided by oligonucleotide architectures based on aptamer technology; its sensitivity can rival with commercial ELISA assay, with the advantage of providing a faster and simpler experimental protocol.

## Results and discussion

### Design, in silico prediction and validation of PSMA-binding oligonucleotide architecture

**Figure 1:**
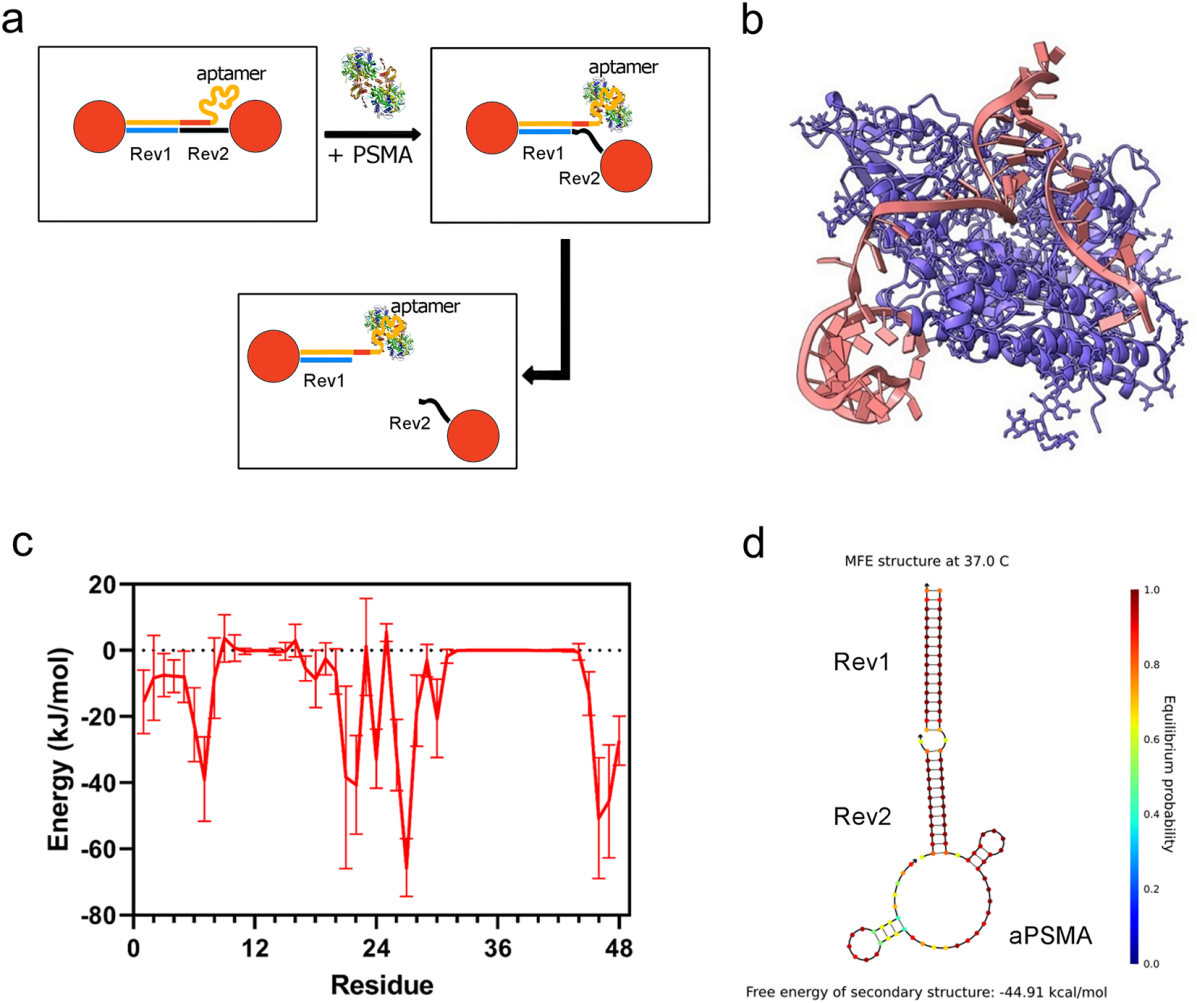
(**a**) Schematic representation of the sensor mechanism of action. (**b**) Docking modelling of the aPSMA ssDNA with the extracellular domain of the PSMA human protein. (**c**) Potential energy contribution of each nucleotide in the aPSMA aptamer sequence to the overall binding energy of the aptamer-PSMA complex. For further information on the energy calculation in silico, see methods section. (**d**) Secondary structures of the aPSMA:Rev1:Rev2 annealed sequences at 37 °C as predicted in silico by NUPACK.

We designed an oligonucleotide architecture, composed of three parts^19^, able to reversibly drive the aggregation of gold nanoparticles. Two thiolated short sequences (Rev1 and Rev2) were conjugated to gold nanoparticles, and the addition of a third oligonucleotide (aPSMA), comprising a previously reported PSMA aptamer^28^ and a portion complementary to strands Rev1 and Rev2 (Fig. 1a and Table S1), leads to the formation of gold nanoparticles aggregates (AuNPs-Aggregates, in this case aPSMA-AuNPs-Aggregates). The structure was designed in such a way that a small number of nucleotides (trigger nucleotides, both underlined and italic in Table S1) in the aptamer sequence is both involved in the binding to the target and hybridized with the complementary sequence Rev2. A control was made by using the same Rev1 and Rev2 and with the sequence comprising the aptamer that was scrambled in the portion not conjugated by them (Control, see Table S1 and Figure S1). Target recognition by the free portion of the aptamer should act as a driving force to promote interaction of the trigger nucleotides with the target protein. This should favour the detachment of the complementary sequence Rev2 (or equivalently a lower melting temperature for the complex), causing disassembly of the Rev2-aptamer structure and thus of the whole aggregate. The first step in the design of such a complex is the identification of the trigger nucleotides. Ideally, these should interact with the protein, to provide the necessary energy gain that compensates, at least partially, the energy loss involved in the detachment from the oligo duplex. At the same time, initial binding by the aptamer should occur also without contribution by these nucleotides and should bring the trigger nucleotides in spatial proximity to the protein, favouring interaction with the protein and de-hybridization. A purely experimental approach would be impractical, requiring to synthesize and test many aptamer-complement couples. For this reason, we set up an in silico protocol obtaining the docking between the aptamer and the reported structure of the recombinant extracellular portion of PSMA (Fig. 1b). Electrostatic and van der Waals contributions to the total binding energy identified the residues 6–7 as the first residues critically involved in the aptamer-protein binding (Fig. 1c). In line with these findings, we designed an extended version of aPSMA aptamer in such a way that the 5’ end of Rev2 hybridizes with residue 7 of the aptamer, and that it hybridizes completely with both Rev1 and Rev2 (Fig. 1D and Table S1). As already stated, the Control strand was designed starting from the aPSMA aptamer binding portion but using a scrambled sequence from base 38 to 78 (base 4 to 48 of the aptamer), and a 3’ tail identical to the one that hybridizes Rev1 and Rev2 (Figure S1 and Table S1). In silico and in vitro melting temperatures indicate that the duplexes are stable at physiological temperature (Figure S2 and Table S2).

### aPSMA-AuNPs-aggregate assembly and characterization

**Figure 2:**
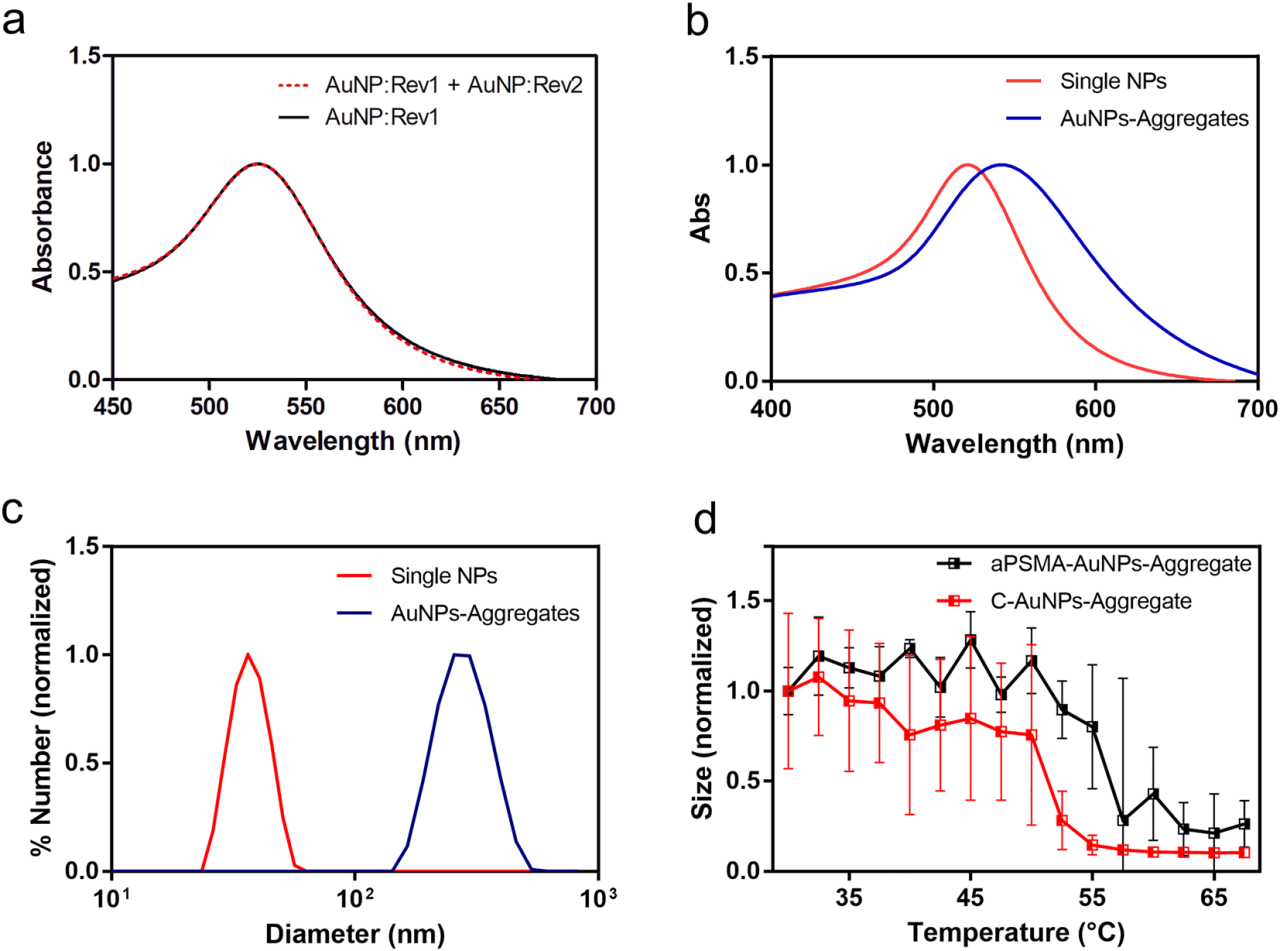
(**a**) Extinction spectra of solutions containing AuNP:Rev1 alone (black curve) or incubated with AuNP:Rev2 (red dashed curve). (**b**) Extinction spectra of aggregated (blue) and of free AuNPs (red). Spectra are normalized with respect to the plasmonic peak value. The AuNPs-Aggregate absorbance spectrum is representative of one preparation, the plasmonic shift can vary depending on the AuNPs-Aggregate batch. (**c**) DLS recording of size (number weighted) distribution for ssDNA-decorated NPs (red) and of aggregated NPs (blue); size curves are normalized at their maximum value. (**d**) Size of aPSMA-AuNPs-Aggregate (black line) and of C-AuNPs-Aggregates (obtained using the Control strand instead of the aPSMA; red line) measured by DLS between 30 and 70 °C. Size is normalized to the value measured at the beginning of each experiment (30 °C).These measurements were repeated in triplicate (n = 3) and error bars are the standard errors.

To investigate the aggregation of AuNPs driven by the oligonucleotides, we synthesized AuNPs of 13 nm in diameter and functionalized them with either Rev1 or Rev2. The obtained nanoparticles show a shifted plasmon peak at 524 nm^29^, a larger hydrodynamic radius and a reduced Z-potential (Table 1); notice that these and all the experiments described in the following are performed in a 1× PBS buffer in order to mimic physiological conditions. The average number of oligonucleotides per particle in our experimental conditions was estimated to be 182 ± 33. Co-incubation of AuNP:Rev1 with AuNP:Rev2, without the linker sequence, does not lead to measurable changes in plasmonic resonance properties, confirming that no non-specific interaction occurs (Fig. 2a).

**Table 1:** Characterization (number-averaged size and Z-potential, as given by the Malvern software) of AuNP nanoparticles and of AuNPs-Aggregates.

	Number-averaged size (nm)	Zeta potential (mV)	Plasmonic peak (nm)
AuNP	13.0 ± 0.6	− 33.6 ± 1.9	520.0 ± 0.6
AuNP:Rev	31.6 ± 0.2	− 53.0 ± 3.4	524.0 ± 0.6
aPSMA-AuNPs-Aggregate	150 ± 7	− 23.4 ± 0.8	539.0 ± 1.0
C-AuNPs-Aggregate	145 ± 55	− 20.7 ± 0.1	540.0 ± 1.5

Measurements were repeated in triplicate (n = 3), the reported uncertainty is the standard deviation. For the two Aggregates indicated in the table, size and zeta potential describe purified aggregates after 24 h of incubation of the two oligo-functionalized nanoparticles with the linker sequences.

Next, we screened different aptamer/Rev ratios for aPSMA-AuNPs-Aggregates assembly, identifying an aptamer/Rev molar ratio of 0.22 as the best one, amongst the tested ones, for both AuNPs-Aggregate formation and hrPSMA response (Figure S3). This formulation was used in all subsequent experiments. The formation of AuNPs-Aggregates, after the addition of the linker sequence, is evidenced by an increase in size for the observed nanosystems in solution, which reaches stability after the first two hours of incubation at 4 °C (Figure S4); note that in Table 1 and in most figures we report the averaged diameters of DLS-detected nanoparticles with averaging weighted by their numbers, since this quantity is more sensitive to the presence of smaller nanoparticles (eventually disassembled or released from the aggregates) than the ones weighted, e.g., by intensity or volume (where the weighting is multiplied by a factor proportional to the third or sixth power of their size, respectively). Monitoring the AuNPs-Aggregate size for 24–72 h after purification suggested a slow but continuous interaction amongst the nanoaggregates even when stored at 4 °C: hydrodynamic radii steadily increased from 150 ± 7 nm to 440 ± 70 nm for aPSMA-AuNPs-Aggregates, and from 145 ± 55 nm to 350 ± 22 nm for the Aggregates obtained using the Control strand (C-AuNPs-Aggregates). The plasmon peak observed in the final purified Aggregate solutions fell within the 539–550 nm range (Fig. 2b) for all the different preparations used in this work, reflecting the aggregation but also the intrinsic variability of the nanostructured system. The trend in variation of plasmonic peak correlates with what  is expected from DLS measurements (Fig. 2c). However, the red shift was smaller than what expected for monolithic AuNPs of similar sizes or than what previously observed by some of us in arrays of closely-packed NPs^30^; this is probably caused by the low density of NPs in the aggregates and therefore their smaller electromagnetic coupling. Indeed, when aggregated, the Au seeds are linked by a dsDNA of about 30 base pairs (around 10 nm). Since both the AuNPs and the DNA are negatively charged, the DNA linker is likely stretched, maximising the surface-to-surface distance and making the electromagnetic coupling between gold seeds only partial. As indicated by previous reports, NP dimers or chains demonstrated a small LSPR shift for NPs of analogous diameters at similar distances^31–33^, e.g. with a peak shift limited to 4–12 nm for 15 nm AuNPs with 10 nm surface-to-surface distance^34^. Also, Au-NPs dimers with surface-to-surface distance close to the diameter of the NPs showed a LSPR shift around 1%^35^. Finally, we verified if the melting temperature of oligonucleotides changed when they were linked with the nanoparticles and inside aggregates. We observed a thermal disassembly profile in line with the one of the single-DNA hybrids (Fig. 2d and Table S2), indicating that AuNP-Aggregate formation and disassembly are driven by the hybridization of the oligonucleotides.

### AuNPs-Aggregates response to hrPSMA

**Figure 3:**
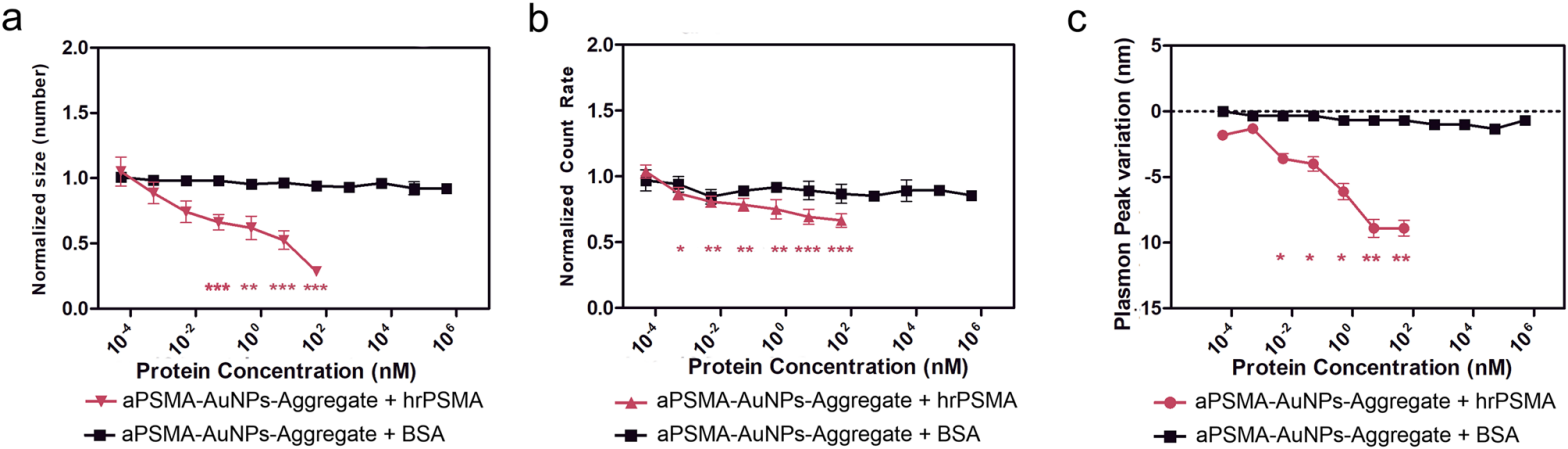
Disassembly of the aPSMA-AuNPs-Aggregate incubated with purified hrPSMA (red graphs) or with BSA (black graphs) in cuvette. Normalized variation in size (number weighted averages, panel **a**) and count rate (**b**) of the aPSMA-AuNPs-Aggregate measured by DLS and relative plasmon peak variation (**c**). For panels **a** and **b** the values reported are normalised to 1 with respect to the situation in the absence of BSA or PSMA. For panel **c** the plasmon peak variation is 0 when the aPSMA-AuNPs-Aggregate are measured in the absence of the target protein. Measurements were repeated n = 3 times for BSA and n = 7 times for hrPSMA. Error bars are standards errors. P values (defined in the experimental section) are for t-tests of aPSMA-AuNPs-Aggregate incubated with the purified protein (hrPSMA or BSA) extracts versus the case without the purified protein.

We investigated the AuNPs-Aggregates response towards PSMA in scenarios of increasing complexity (human recombinant PSMA -hrPSMA- and PSMA in small extracellular vesicles -SEVs- or in their extracts) using DLS and extinction measurements. DLS evidenced a reduction in size of nanoaggregates in the presence of hrPSMA, significant at hrPSMA concentrations greater than 50 pM (*p* < 0.05, Fig. 3a). Also, the count rate in DLS measurements decreased significantly at hrPSMA concentrations higher than or equal to 500 fM (*p* ≤ 0.05, Fig. 3b): in DLS, count rate is a function of both number and size of the analysed particles; during the disaggregation process, the number of nanoparticles increases, while the scattering intensity of each generated nanoparticle decreases. Disaggregation of a single aggregate in N smaller nanoparticles causes a decrease in nanoparticle radius proportional to ${N}^{-\frac{1}{3}}$. Since the scattered intensity is proportional to the 6th power of the radius, it is expected that the whole process of disaggregation of an aggregate in N smaller particles causes a scattering intensity scaling as ${N}^{-1}$, therefore decreasing, as observed in our experimental conditions. The observed decrease in size and in plasmon resonance for the aPSMA-AuNPs-Aggregates upon hrPSMA recognition happens within the first minutes (Figure S5a). Control experiments carried out with C-AuNPs-Aggregates did not show any significant trend (Figure S6). We checked if aPSMA-AuNPs-Aggregates could disassemble in the presence of other proteins using BSA: neither aPSMA-AuNPs-Aggregates nor the C-AuNPs-Aggregates showed significant changes in size when incubated with BSA at concentrations ranging up to 500 μM, a concentration similar to the ones of proteins in human plasma (Figure S6), indicating negligible non-specific interaction with serum proteins up to the physiological level.

When evaluating the disassembly of the system by measuring plasmon-peak shift of AuNPs-Aggregates, we observed a significant hypsochromic shift of the aPSMA-AuNPs-Aggregates starting from a hrPSMA concentration of 5 pM (*p* < 0.05), but not in control experimental settings (Figure S6), in qualitative agreement with what observed by DLS (Fig. 3c and S5b). However, the plasmon peak does not match the one of single nanoparticles even at the highest tested concentration (hrPSMA 50 nM), the maximum shift being 8.5 nm (from 550 to 541.5 nm), probably because not every aggregate is completely disassembled (Figure S7a,b). The number-weighted average size for aPSMA-AuNPs-Aggregate at 50 nM hrPSMA (91 ± 27 nm, corresponding to the last red triangle in Fig. 3a) indicates the release of smaller AuNPs-aggregates. UV–Vis measurements are influenced by a small number of residual large aggregates, whose higher plasmon-resonance intensities dominate the resulting UV–VIS spectrum (Figure S7a,b)^36^. Indeed, DLS intensity-weighted size distributions display two peaks, one probably due to aPSMA-AuNPs-Aggregates, and one at lower sizes compatible with small aggregates, whose area starts very small but increases at higher hrPSMA concentration (Figure S7b,b). Also in this case, no peaks compatible with single particles seems to be present; however, the distribution of the diameters of the nanoparticles weighted with their numbers are actually inferred from data depending on the intensity of the scattering (proportional the sixth power of the size, as specified above), and data on the smallest nanoparticles can be lost due to the lower signal-to-noise ratio.

The measurements of the disassembly of aPSMA-AuNPs-Aggregates as performed here produced results different from the controls at a statistically significant level starting from sub- or low- pM concentrations of the analyte, depending on the detection method. Statistical significance does not compare directly with an equilibrium constant, which could be difficult to measure or even to define since the multimerization in "reagents" and "products" is not perfectly defined. Indeed, disaggregation might not be complete, and in each Aggregate there are many aptamers, the more aptamers the bigger the aggregate. In any case, this fact could also be responsible for a higher sensitivity: aptamer “multivalency” has already been related to an increased binding avidity with respect to the monomeric aptamer^37^. In our case, the increased binding affinity is traduced in a higher Rev2-AuNPs detachment. In addition, we must consider that the aptamer-protein binding reaction can be described as a dynamic equilibrium. When following the kinetics of the reaction, we observed a fast Aggregates disaggregation, occurring within minutes after the addition of the analyte, and a kinetics for the assembly of nanoparticles in Aggregates that was slow with respect to the time window of the experiment (Figure S5A compared with Figure S4); these observations make the disaggregation of the nanoarchitecture irreversible for our practical purposes.

### AuNPs-Aggregates response to PSMA positive SEVs and their protein extract.

**Figure 4:**
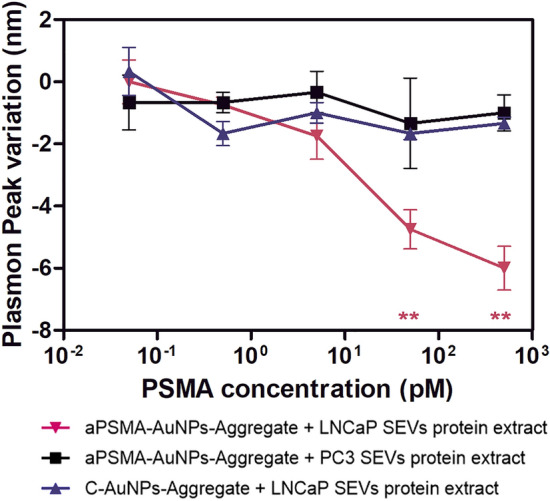
aPSMA-AuNPs-Aggregates responded to exosomal-derived PSMA when incubated with high concentrations of protein extract of PSMA positive SEVs (red line). On the x-axis are reported equivalent PSMA concentrations calculated as if PSMA be 0.51% in weight of the total protein extract for PC-3 SEVs protein extract as well (5 pM corresponds to 100 ng/ml of protein extract; its analysed concentrations ranged from 1 ng/ml to 10 μg/ml). *P* values (**p < 0.01) are for t-tests of aPSMA-AuNPs-Aggregate incubated with SEVs extracts versus the case without SEVs extract. Plasmon peak variations are 0 when the AuNPs-Aggregates are analysed in the absence of extracts. All measurements were repeated in triplicate (n = 3) and error bars are standard errors.

AuNPs-Aggregates were incubated with increasing concentration either of intact LNCaP (PSMA +) and PC-3 (PSMA-) SEVs, or of their protein extracts. The purified SEVs were preliminarily characterized in terms of size (Figure S8d,e) and protein content (Figure S8a, S8c, S8c and S9), finding results in agreement with what was expected for the specific SEVs. In particular, PSMA quantification through an ELISA assay on the extract from LNCaP SEVs indicated a PSMA concentration corresponding to 0.51% of the total protein content in weight (Figure S8F). We will not discuss thoroughly the results obtained by DLS measurements, because the high scattering of SEVs affects these results, and the reproducibility of the results obtained using protein extracts seems to be decreased. This is likely due to the confounding effect of surfactants contained in the medium used to produce extracts (RIPA buffer), that might hamper the measurement either by aspecific interaction or by optical interference. Although SEV preparations were highly diluted before use, it is reasonable to consider that the residual presence of surfactants in such preparations could affect their dynamic properties (see Figure S10). Considering plasmon peak variations, aPSMA-AuNPs-Aggregates optical properties did not show any significant change upon incubation with untreated whole PSMA + SEVs, like for the controls (aPSMA-AuNPs-Aggregates with PSMA- SEVs or C-AuNPs-Aggregates with PSMA + SEVs, see Figure S11). Conversely, incubation of LNCaP SEVs protein extract (but not PC-3 SEVs extract) with aPSMA-AuNPs-Aggregates (but not with C-AuNPs-Aggregates) produced a significant plasmon peak variation starting from protein concentrations corresponding to a PSMA one of 50 pM (*p* < 0.01, t-test; Fig. 4). The highest tested PSMA concentration was 500 pM due to the yield of protein purification from SEVs. The fact that no significant changes have been observed with PC-3 SEVs extract is another demonstration of the stability/unresponsiveness of the system in the presence of proteins different from PSMA: considering that the protein concentration of SEVs extracts is approximatively 200 times the one of PSMA, no changes have been observed up to 10 μg/ml of various proteins. The apparently contrasting result between SEVs and their extracts is easily rationalizable considering the aptamer-protein binding geometry, as reconstructed in Fig. 1b. The aptamer was originally developed starting from the recombinant hrPSMA, which consists only of the extracellular portion of the protein, and interacts also with domains that are in close contact with the SEV membrane. Thus, the recognition of the native PSMA protein by the aptamer in intact SEVs could be hampered by steric reasons. The same does not apply when the sensing procedure is performed on SEVs extracts, where we obtained significant changes in the low picomolar range. Therefore, we envision the possibility of sensing PSMA in whole SEVs, without the need for protein purification, by employing engineered aptamers that specifically interact with extracellular portions of the protein far from the plasma membrane^38^.

## Discussion

In conclusion, we designed and realised a nanostructure based on aggregated gold nanoparticles that disassembles in the presence of a target protein (in this paradigmatic study, PSMA) at picomolar concentrations. The system is based on a known aptamer, but we described an in silico method that can help to obtain switchable duplexes from already-developed aptamers. A sensor based on this can be used on raw protein extracts, without the necessity for complex isolation and purification procedures of the target biomolecule. In the present implementation, it detected PSMA (recombinant or extracted from SEVs) at concentrations rivalling the sensitivity of ELISA or Western-blot assays. The approach does not rely on enzymatic amplification or protein purification, and in this sense it is superior to current ELISA assays in terms of required time and sample manipulation. This opens the possibility to develop nanosensors where their disassembly is monitored by simple optical setups (UV–VIS spectroscopy and scattering intensity), and to promote their diffusion in the clinical diagnostic environment for routine screening analyses, where robustness and accessibility of the analytical approach are of critical importance. For instance, this sensor could be applied for the detection of PSMA in urinary exosomes of prostate cancer patients, whose detection seems to be highly specific for the disease^39,40^, providing a specific test even less invasive than on a blood sample. The proposed sensing mechanism offers margins of improvement in the development of aptamer-switching structure based nanosensors also by the employment of nanoparticles with different properties (materials, sizes and/or morphologies) or with alternative aptameric sequences and duplex designs. Most importantly, the development of this platform is based on a rational approach that starts from in silico evaluations and that can be easily applied to the development of different nanosensors without complex experimental approaches. The flexibility provided by aptamer technology has the potential to be extended to other types of pathologies that can be identified by univocal biomarker by changing the responsive aptameric sequence. Interestingly, oligonucleotide architectures that disassemble could be adapted in the future in implementations where disassembled reporters are actively separated from the unassembled ones, either *in-vivo* (e.g. by the renal system) or *ex-vivo.* A pertinent example of this concept is evident in the recently developed *ex-vivo* activatable diagnosis systems^41^, which rely on the release of renal clearable nanoparticles. This application makes therefore the disassembly irreversible and should strongly increase our diagnostic abilities^41^.

## Experimental Section

### Materials and Instrumentation

Oligonucleotides (Table S1) were purchased lyophilized from IDT™ and suspended in millipore water, no DNA sequencing was performed in this study. PC-3 and LNCaP cell lines were obtained by ATCC®. No animals or humans were involved in the study. The human recombinant PSMA (hrPSMA) purified protein was acquired from Sinobiological. The antibodies are: antiPSMA (Invitrogen, 1H8H5), antiCD63 (Invitrogen, 10618D) and antiTOM20 (Santa Cruz Biotechnology®, sc-17764). Protein quantification was performed with the Bradford Reagent (Sigma-Aldrich®). The reagents used in Western Blot were purchased by Bio-Rad. Anti-PSMA ELISA assay was purchased by Elabscience® and results were measured with Infinite ® PRO 200 (TECAN). Chemicals used in this study were the following: Gold(III) chloride trihydrate (Sigma-Aldrich®), tri-Sodium citrate dihydrate (VWR) and Tris(2-carboxyethyl)phosphine hydrochloride (TCEP) (Sigma-Aldrich®). DLS (Malvern Zetasizer Nano ZS) and UV–Vis spectra (Cary 3500 UV–Vis) were used for DNA architectures and Nanoparticle characterisation. Attenuation and measurement duration were set on automatic mode on the DLS for size and count rate measurements, with a 173° backscatter measurement angle, and for Zeta potential measurements. Particle sizes reported are the recorded “Number Mean” calculated by the Malvern software, which is the number-weighted average of the average size characterizing the various peaks in the number-weighted distribution of detected particles diameters.

### Cell Culture and small extracellular vesicle (SEV) isolation and characterisation

PC-3 and LNCaP cell lines were cultured in respective media at 37 °C and 5% CO_2_. For SEVs collection, cells were harvested for 48 h in serum-depleted media before proceeding to exosome isolation by ultrafiltration method. Cells and cellular debris were removed by centrifuging at 3,000 xg for 30 min at 4 °C. Then, supernatant was pelleted at 10,000 xg for 1 h at 4 °C to remove microvesicles and the resulting supernatant was filtrated (SterilFlip® 0.22 μm). SEVs were concentrated in PBS (1 X, pH 7.4) with Amicon® Ultra-15 10KDa and stored at -20 °C. SEVs size was measured with DLS. SEVs protein content was extracted in RIPA buffer for 30 min with additional sonication every 10 min. SEVs protein extract was finally concentrated on Amicon® Ultra 0.5 mL filters with a cut-off of 3 KDa. SEVs protein extract was quantified with the Bradford Reagent. SEVs were characterised by Western Blot to verify the presence of the exosomal marker CD63, the absence of the mitochondrial marker TOM20, and the presence of PSMA in LNCaP-derived SEVs. Human PSMA ELISA assay was performed, in triplicate and as specified by the producer, in LNCaP-SEVs extracts to determine PSMA concentration and in PC-3 SEVs extracts, as negative control, to confirm PSMA absence.

### In silico prediction of PSMA-binding aptamer residues

 We developed an in silico protocol based on freely available tools to predict which nucleotides of the aptamer are in contact with the target protein. The secondary structure of the aptamer was predicted using the NUPACK^42^ webserver, imposing physiologic salt conditions ([Na^+^] 0.150 M, [Ca^2+^] 25 mM) at 37 °C. The RNA sequence homolog to that of the PSMA-binding aptamer (T nucleotides substituted with U), together with the base pairings predicted by NUPACK, were fed to RNAcomposer^43^. The resulting structure file was then edited to transform U residues back to T residues and the ribose sugar to deoxyribose, and local minimization was performed with NAMD software package^44^ exploiting CHARMM36 force field^45,46^. The obtained structure was submitted, along with the crystallographic structure of the hPSMA extracellular domain (PDB code: 5O5T), to the HADDOCK webserver^47^, which performs flexible docking between the aptamer and the hPSMA extracellular domain. HADDOCK outputs several structural models of the complex, ranked according to an energy score and grouped in aggregates of similar configurations. The aggregate with the best score was selected^47^ and a further molecular dynamic simulation with explicit solvent was performed starting from the representative structure on a timescale of 12 ns. Simulation was performed in NPT ensemble (T = 300 K, *p* = 1 bar) in a cubic box of 108 Å^3^ by using NAMD package^44^ and exploiting a standard parameter setup^48^. CHARMM36 force field^45,46^ was employed for protein, DNA and counterions to neutralize the cell, whereas TIP3P model^49^ was used to treat water molecules. The energy contribution to the binding was evaluated for each nucleotide by measuring average and standard deviation of the nucleotide-protein interaction energy for the last 4 ns of the simulation^48^. The interaction energies between each nucleotide and the protein were evaluated exploiting NAMD^48^. The long-range part of the electrostatic interactions was neglected whereas the distance cut off for non-bonded interactions was set to 12 Å, and a switching function was applied to smooth interactions between 10 and 12 Å.

### Nucleotide sequences design and characterisation

We designed two groups of sequences (Figs. 1 and S1, Table S1). A first group includes an aptamer responsive to PSMA and is composed of Rev1, Rev2 and aPSMA. A second group used as negative control consists of Rev1, Rev2 and Control sequences. The aPSMA strand was adapted from a previously reported PSMA aptamer^28^, with the addition of a tail complementary to part of Rev2 and Rev1. The Control sequence is composed by the same tail and by a scrambled sequence of the aptamer. Interaction between sequences was analysed in silico with NuPACK online tool to evaluate their secondary structure and thermal stability^42,50^. Thermal stability was calculated between 30 and 70 °C considering a saline concentration of 150 mM for NaCl and 2.5 mM for Mg^++^ and a nucleotide concentration of 100 nM. Melting temperature was considered as the temperature at which the concentration of the paired strands decreased from 100 to 50 nM. In silico thermal stability of the two groups of sequences was analysed considering first the interaction of aPSMA (or Control) with the full sequence of Rev1 and Rev2 strand, followed by the analysis of the melting temperature between aPSMA, Rev1 and the portion of Rev2 strand without the nucleotides complementary to the initial part of the aptamer (Rev2-short, Table S1). Thermal stability of the annealed sequences was confirmed in vitro by UV–Vis absorbance measurements monitoring the hyperchromicity at 260 nm, exploiting the 1.5 folds increase in molar extinction coefficient of oligonucleotides^51^ occurring upon transition from double to single strand. AntiPSMA or Control sequences, with Rev1 and Rev2, were first annealed heating at 95 °C for 2 min, and then slowly cooled down from 94 °C to 12 °C with a rate of -2 °C per minute in Annealing Buffer (50 mM Tris–HCl, pH 7.5, 150 mM NaCl, 25 mM MgCl_2_). Then, melting curves of the annealed strands were recorded (using a concentration of 100 nM for each nucleotide) from 30 °C to 70 °C at a ramp rate of 1 °C per minute; melting temperature was calculated by looking for the maximum value of the first derivative of the absorbance with respect to the temperature.

### AuNPs synthesis and functionalisation

13-nm diameter AuNPs were synthetized as previously reported^19^ and characterised by DLS and by absorbance measurements. AuNPs concentration was determined by plasmonic peak intensity and molar extinction coefficient (3.60 × 10^8^ M^-1^ cm^-1^ for AuNPs of 13 nm diameter)^19^. AuNPs were decorated with Rev1-thio, Rev2-thio (sequences functionalised with thiol modifier C3 or C6, see Table S1). Thiol-modified oligonucleotides were deprotected with a tenfold molar excess of TCEP in a Tris Acetate 25 mM ph 5.2 buffered solution for one hour at 25 °C, then incubated overnight at room temperature with nanoparticles solution using a DNA/AuNP ratio of 1000. The day after, NaCl 1 M and Tris acetate 500 mM pH 8.2 were slowly added to the DNA-NPs solution up to a final concentration of 300 mM NaCl and 5 mM Tris acetate pH 8.2; the mixture was incubated overnight at room temperature. AuNPs were purified by centrifuge at 16,100 x*g* for 15 min, removing supernatant and suspending pelleted nanoparticles. This step was repeated three times; AuNPs were finally suspended in NaCl 300 mM, Tris acetate 5 mM, pH 8.2 buffer solution. Functionalised AuNPs were characterized by DLS for size and zeta potential and by UV–Vis spectra for plasmonic peak determination DLS measurements for single and aggregated-AuNPs were performed diluting stock solutions by a factor of 10 in PBS for size and in a 10 mM NaCl solution for zeta-potential determination. DNA/AuNPs ratio was estimated by subtracting from the total amount of DNA used in the functionalisation reaction the unreacted DNA strands, whose quantity was estimated by absorbance reading at 260 nm from all the supernatants obtained during the purification steps.

### Aggregate preparation and characterization

AuNPs-Aggregates were prepared as previously reported^19^. Nanoparticles that expose complementary sequences and the linker sequence were incubated in this way (reaction mixture): AuNPs:Rev1, AuNPs:Rev2 with aPSMA (aPSMA-AuNPs-Aggregates) and AuNPs:Rev1, AuNPs:Rev2 with Control sequence (C-AuNPs-Aggregates). The optimal formation of aPSMA-AuNP-Aggregates was first screened incubating AuNP:Rev1 and AuNP:Rev2 with increasing concentration of the aPSMA linker sequence at the following concentrations: 1 nM, 10 nM, 100 nM, 1 μM and 10 μM. These firsts AuNPs-aggregates were prepared in a 1 × PBS buffer, pH 7.4, overnight at 4 °C while stirring and characterised without further purification. For the following PSMA-response experiments, the aPSMA and Control sequences were added in the reaction mixture at a final concentration of 100 nM. Only in this case, the kinetic of AuNP-Aggregate formation was monitored for 4 h. In all other experiments, these solutions were incubated overnight at 4 °C while stirring and the AuNPs aggregation reaction was performed in a 300 mM NaCl, 5 mM Tris acetate pH 8.2 buffer solution. The day after, aggregates were purified centrifuging the solution at 800 *xg* for 1 min and resuspended in 150 mM NaCl 5 mM Tris acetate pH 8.2 buffer solution. Aggregates were characterised by DLS for size and zeta-potential and with UV–Vis absorbance measurements for plasmon peak determination. Thermal stability was measured incubating aggregates solutions in DLS at different temperatures (from 30 to 70 °C).

### In-cuvette hrPSMA response

AuNPs-Aggregate response to PSMA was tested with human recombinant PSMA (hrPSMA) purified protein. Firstly, we characterized size and plasmon peak variations incubating the unpurified aggregates (where the aPSMA linker sequence ranged from 1 nM to 10 μM) with hrPSMA at a concentration of 50 pM for 30 min at 37 °C. For the next sensing experiments, hrPSMA was diluted in PBS (1 X, pH 7.4) at concentrations ranging between 50 fM and 50 nM. AuNPs-Aggregate response to hrPSMA was obtained incubating, in PBS, the protein solution with the aggregate(final optical density was maintained between 0.18 and 0.23, measured at λ = 540 nm). Aspecific interaction was evaluated using, with the same experimental protocol, BSA at concentrations from 50 fM to 500 μM (which corresponds to the physiological albumin blood concentration). The process was preliminary monitored in time for 20–22 min by DLS to determine the kinetics of the reaction. Variation in size and plasmon peak properties was characterised by DLS and UV–Vis after the addition of the analyte. Variations in the plasmonic maxima of aggregates were calculated by looking for the zero of the first derivative of the absorbance with respect to the wavelength values.

### Response to PSMA positive SEVs

AuNPs-Aggregate response to LNCaP and PC-3 SEVs was investigated incubating the AuNPs-Aggregates with increasing concentration of intact exosomes or with exosomal proteins extracted in RIPA buffer. Final optical density was maintained between 0.17 and 0.36 at λ = 540 nm, identical when comparing positive and negative exosomes. The AuNPs-Aggregates were incubated at 37 °C for 30 min in PBS (1 X, pH 7.4) with different concentration of exosomes or of their extract (corresponding to PSMA protein concentrations in LNCaP exosomes or their extract ranging from 1 ng/ml to 10 μg/ml). Intact SEVs or SEVs protein extracts were diluted in PBS buffer (1X, pH 7.4). AuNPs-Aggregate sensing response was determined monitoring the variation in plasmonic shift of AuNPs-Aggregates.

### Statistics

All data are reported as mean ± standard error unless specified otherwise. The data set relative to the aPSMA-AuNPs-Aggregate formation and hrPSMA response pre-screening was analysed with 2-way ANOVA test with Bonferroni post-hoc tests. All the others data sets were analysed with t-tests. Significance was attributed with a P value less than 0.05. In figures, significance is represented as follows: *p* < 0.05 is *, *p* < 0.01 is ** and *p* < 0.001 is ***.

### Supplementary Information

Supplementary Information.

## Data Availability

The datasets analysed during the current study are available in the Uniprot repository, PDB code: 5O5T (https://www.rcsb.org/structure/5O5T#entity-1).
