# Supplementary material for: Aptamer-based gold nanoparticle aggregates for ultrasensitive amplification-free detection of PSMA
Source: Sci Rep. 2023 Nov 15;13:19926. doi: 10.1038/s41598-023-46974-4 (PMC10651859; doi:10.1038/s41598-023-46974-4)
Supplement: Supplementary file 1 — Supplementary Information. [file 41598_2023_46974_MOESM1_ESM.pdf]

Supplementary Information

Table S1: Complete list of the DNA sequences used in the present study. “thiol” indicates the presence of a C3 thiol modifier (at 3’) or C6 thiol modifier (at 5’). Bold letters highlight Rev1 and its complementary sequence; underlined letters highlight Rev2 and its complementary sequence. The part in italic corresponds to the published PSMA aptamer or to its scrambled Control sequence.

**Published sequences**	
PSMA aptamer	5’ – *GCG TTT TCG CTT TTG CGT TTT GGG TCA TCT GCT TAC GAT AGC AAT GCT* –3’
**Sequences used in the study**	
Rev1-thio	5’– **CAC AGA TGA GTC ACA G** – thiol – 3’
Rev2-thio	5’ – thiol – AAA ACG CCG CCC TT – 3’
Rev1	5’– **CAC AGA TGA GTC ACA G** –3’
Rev2	5’ – AAA ACG CCG CCC TT – 3’
Rev1-NH_2	5’ – amine – **CAC AGA TGA GTC ACA G** –3’
Rev2-NH_2	5’ –AAA ACG CCG CCC TT – amine – 3’
Rev2-Short	5’- CGC CCT T -3’
aPSMA	5’ – **CTG TGA CTC ATC TGT G**AA GGG CG*G CGT TTT CGC TTT TGC GTT TTG GGT CAT CTG CTT ACG ATA GCA ATG CT* – 3’
Control	5’ – **CTG TGA CTC ATC TGT G**AA GGG CG*G CGT TTT ATA TGT TAC CGT CTA AGT ACT CTG TTC CGC GCC GTG TGT TG*– 3’

Table S2: comparison between melting temperatures (T_{melt}) in silico, in vitro, and of AuNPs-aggregates, with a final DNA concentration of 100 nM.

**Sequence**	***In silico* T_{melt} (NuPACK)**	***In vitro* T_{melt} (from 260 nm abs)**	**Nanosaggregates T_{melt} (DLS)**
antiPSMA	56 °C	56 °C	52.5 °C
Control	56 °C	59 °C	50 °C

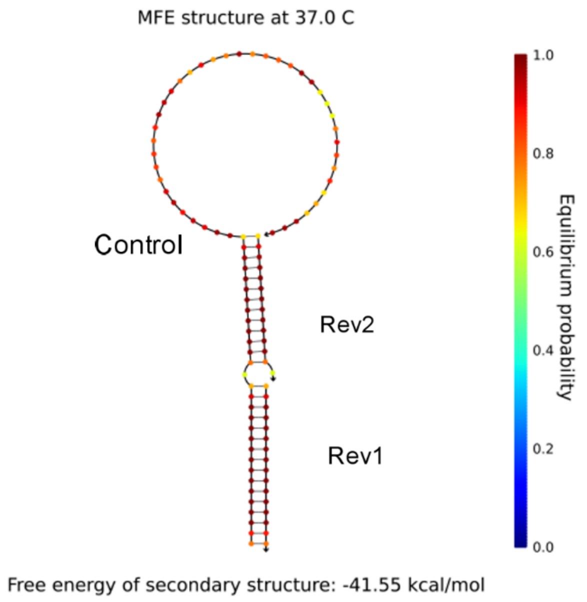

Figure S1. Secondary folding structures of the Control:Rev1:Rev2 group of sequences at 37°C as predicted in silico by NUPACK.

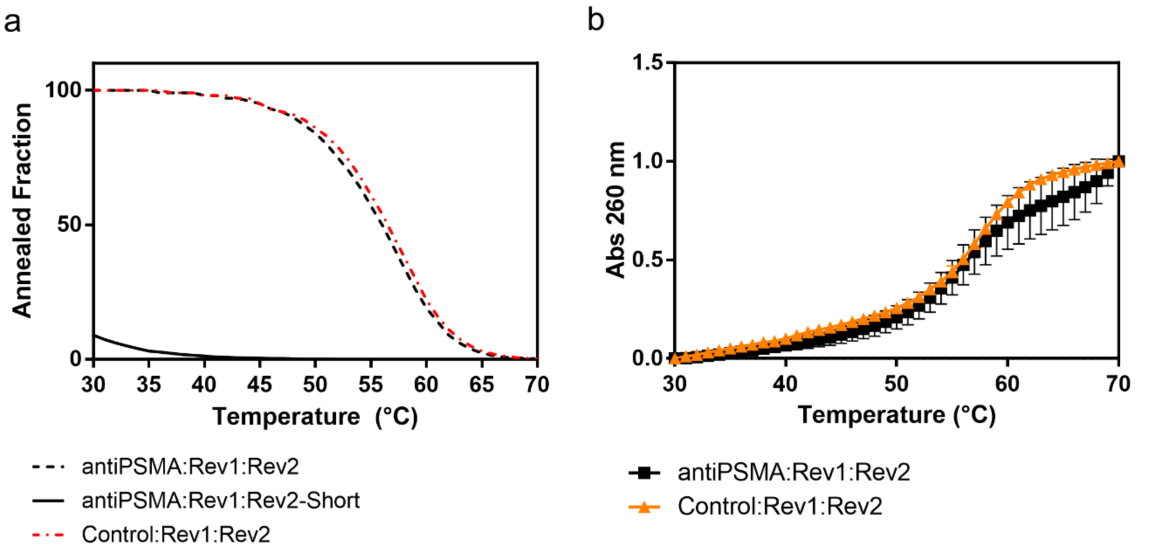

Figure S2. (**a**) Melting temperature in-silico analysis, performed with NUPACK, of Control:Rev1:Rev2 (red dotted line) and antiPSMA:Rev1:Rev2 annealed sequences (black dotted line) without the target protein. We report as a black solid line a simulated thermal stability curve for of a complex with a version of Rev2 without the seven nucleotides that hybridize with the aptamer (Rev2-Short), simulating the conditions of the antiPSMA:Rev1:Rev2 complex when PSMA is present. (**b**) Measured melting curves of antiPSMA:Rev1:Rev2 (black) and Control:Rev1:Rev2 (orange) at a concentration of 100 nM. Absorbance values are recorded at a temperature between 30°C and 70°C and are normalized to 1 at the highest considered temperature (70°C). Measurements in panel **b** were repeated in triplicate (n=3) and error bars indicate the standard error. In panel B, the slow underlying growth of the curves could be caused by possibly hybridized nucleotides in the binding (i.e. single strand) portion of the aptamer, leading to an uneven baseline.

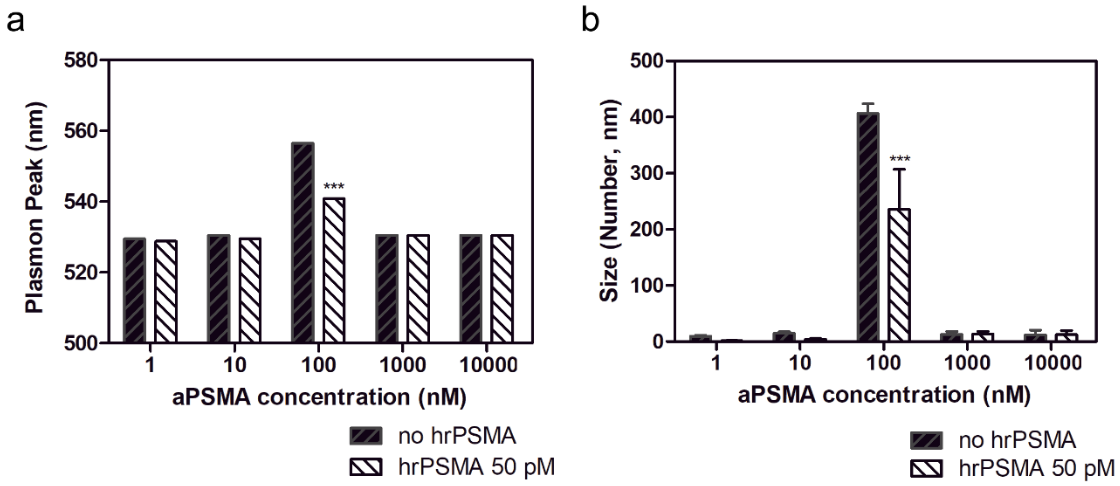

Figure S3. The effect of the linker sequence concentration was tested in the AuNPs-Aggregate formation. Variations in plasmonic resonance (**a**) and in size (**b**) for aPSMA-AuNPs-Aggregates with respect to single nanoparticles were registered incubating AuNP:Rev1 and AuNP:Rev2 solutions (DNA concentration: 455 nM for Rev1 and Rev2) with the aPSMA strand at increasing concentration, ranging from 1 to 1000 nM (black bar). We identified an aptamer/Rev molar ratio of 0.22 (corresponding to an aPSMA concentration of 100 nM in the reaction conditions) as the best ratio for AuNPs-aggregate formation among the tested conditions. Lower or higher aptamer/Rev ratios produced smaller nanostructures or single non-interacting nanoparticles. Then, the obtained aPSMA-AuNPs-Aggregates were tested with 50 pM hrPSMA concentration (white bar) in order to detect the aPSMA-AuNPs-Aggregate that responded to hrPSMA with the highest variation in size. Aggregates obtained using 100 nM aPSMA provided the best response in terms of changes in size ($p<0.001$) and plasmon peak properties, evidencing a peak shift of 16 nm ($p<0.001$). In all the other tested aPSMA-AuNPs-Aggregates conditions (different linker concentration and incubation with hrPSMA), the plasmon maximum variation always falls in the 0 to 1.3 nm range. All measurements were repeated in triplicates (n=3) and error bars are standard errors.

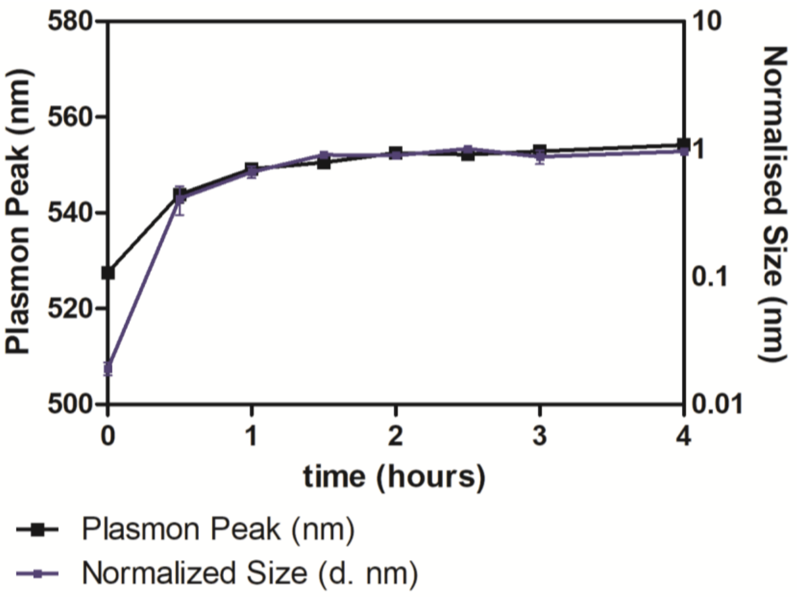

Figure S4. AuNPs-Aggregate formation was monitored for the first 4 hours immediately after the addition of the linker sequence to a mixture of AuNP:Rev1 and AuNP:Rev2 nanoparticle solutions at 4 °C (no purification step was done). Variation in number-averaged size (blue line) and plasmonic shift (black line) indicated that the AuNPs assemble into Aggregates within the first hours. Size is normalized to 1 at the last recorded measure (time=4 h). All measurements were repeated in triplicate from the same AuNPs preparation (n=3) and error bars indicate the standard error.

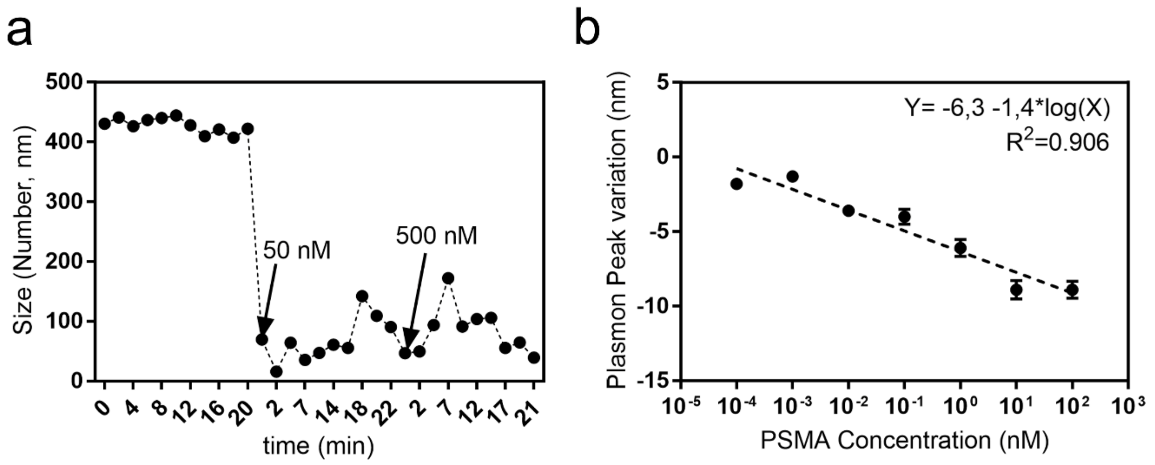

Figure S5: (**a**) Representative disaggregation kinetics of aPSMA-AuNPs-Aggregate at DLS upon additions of hrPSMA. AuNPs-Aggregates size is stable in time (here recorded for 20 minutes), with a diameter ranging from 407.4 to 444.5 nm. Then, the same AuNPs-Aggregate solution was incubated with hrPSMA at a final concentration of 50 nM and of 500 nM. The aPSMA-AuNPs-Aggregate showed a rapid reduction in size (number weighted average) immediately after the addition of the analyte. When incubated with hrPSMA at a concentration of 50 nM, the observed sizes ranged from 16.1 to 149.1 nm up to 22 minutes from the addition of the biomarker. When the hrPSMA was added to a final concentration of 500 nM the measured nanostructures sizes remained in the same range (39.5-172.4 nm) in the next 21 minutes. These data indicate that the release of single AuNPs and/or of smaller aggregates is fast, and that this phase can be followed by a slower dynamics with re-aggregation and further disaggregation. (**b**) Nonlinear regression analysis of the plasmon peak variation trend of the aPSMA-Au-Aggregate incubated at increasing concentration of hrPSMA. The nonlinear regression analysis was obtained considering a logarithmic X axis and a linear Y axis. Measurements were repeated n=7 times and error bars are standard errors.

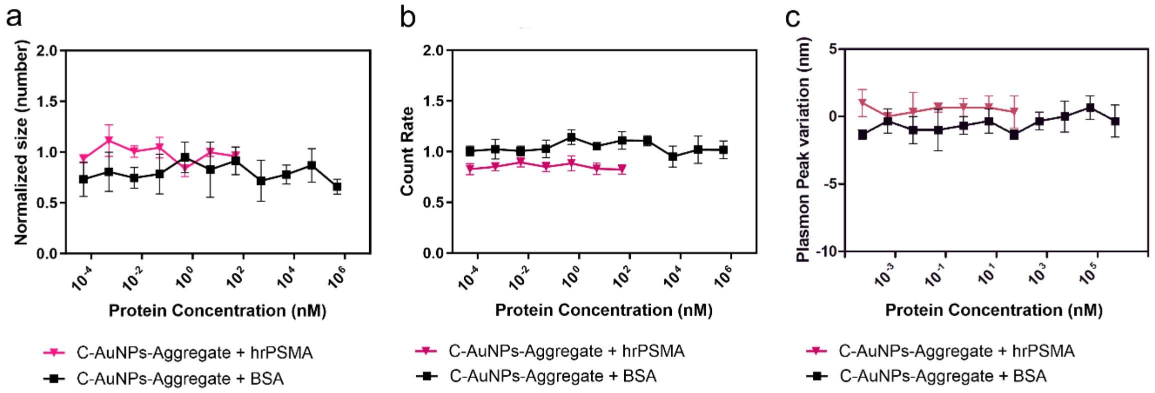

Figure S6. Response of C-AuNPs-Aggregates incubated with purified hrPSMA or BSA proteins in cuvette. (**a**) Normalized variation in size (number-weighted average) of the C-AuNPs-Aggregate as a function of hrPSMA (red) and BSA (black) concentration. (**b**) Normalized variation in count rate of the C-AuNPs-Aggregate as a function of hrPSMA (red) and BSA (black) concentration. (**c**) Relative plasmon peak variation of C-AuNPs-Aggregate at different hrPSMA (red) and BSA (black line) concentrations with respect to the AuNPs-Aggregate without the target protein. For panels **a** and **b**, the reported values are normalised to 1 with respect to the aPSMA-AuNPs-Aggregate in absence of BSA or PSMA (Protein concentration = 0 nM). For panel **c**, the plasmon peak variation is considered to be 0 when the aPSMA-AuNPs-Aggregate are measured in absence of the target protein. All measurements were repeated in triplicate (n=3) and error bars are standard errors.

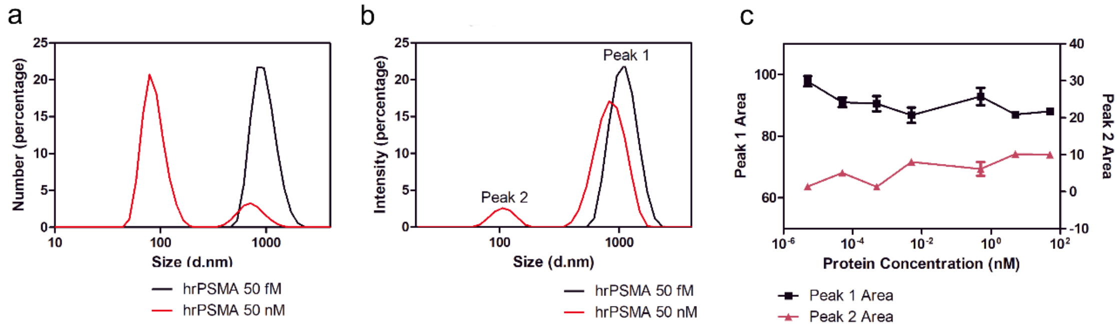

Figure S7. Representative number weighted (**a**) and intensity weighted (**b**) size distributions recorded by DLS from the same aPSMA-AuNPs-Aggregates solution upon addition of the lowest (50 fM, black line) and the highest (50 nM, red line) hrPSMA total concentration, as tested in our experimental setting. (**c**) Dependence on hrPSMA concentration of the areas of the two main peaks in the DLS-measured intensity-weighted size distributions. In these two sets of measurements (n=2, error bars are standard errors) we observed a higher peak (Peak 1 in the graph) corresponding to aPSMA-AuNPs-Aggregates, and a smaller one (Peak2) corresponding to released smaller AuNPs-aggregated and/or single AuNPs, as shown in panel **b**. The bigger aPSMA-AuNPs-Aggregates peak contains 98.5±1.3 % of the distribution without the presence of the hrPSMA and its abundance decreases to 88.1±0 % at the highest tested hrPSMA concentration. The percentage of Peak2 increases from 1.3% to 10% at the highest hrPSMA concentration used in the sensing reaction.

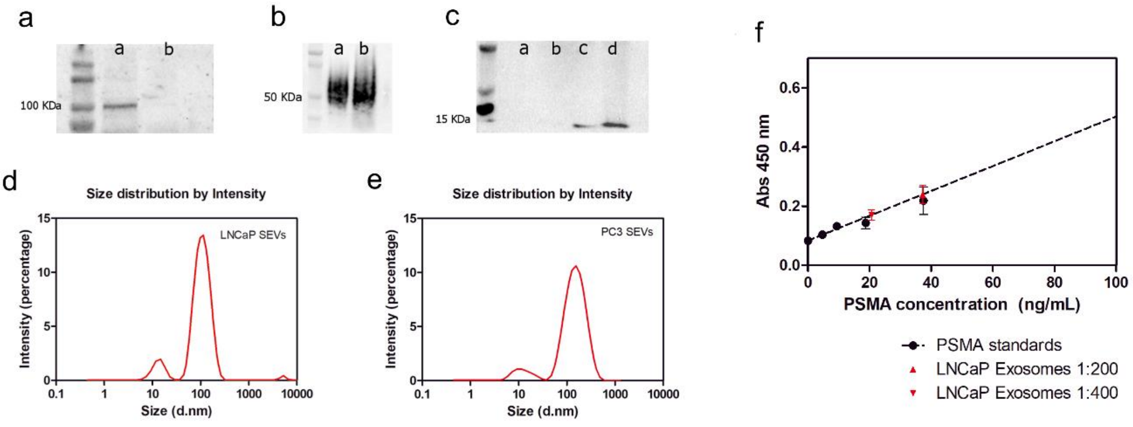

Figure S8. Characterization of small extracellular vesicles (SEVs) extracted from the culture media of LNCaP and PC-3 cell lines (**a**, **b**, **c**) Western Blots for isolated SEVs characterisation. Images of the uncut membranes are reported in Figure S9. The lane most at left contains the weight scale from the Precision Plus Protein Dual Color Standards (Bio-Rad®) ladder. (**a**) PSMA blotting resulted positive for LNCaP SEV (lane a) and negative for PC-3 SEV (lane b); (**b**) Exosomal marker CD63 presence was detected both in LNCaP SEVs (lane a) and PC-3 SEVs (lane b); (**c**) mitochondrial marker TOM20 was not present in LNCaP and PC-3 isolated SEVs (lanes a and b) and its blotting was positive in LNCaP cells (lane c) and PC-3 cells (lane d) extracts. (**d**,**e**) Representative size intensity-weighted distributions of LNCaP (**d**) and PC-3 (**e**) isolated SEVs measured with DLS. Size as determined by DLS measurements was 200±73 nm for PC-3 and 115±90nm for LNCaP-derived SEVs (Intensity-weighted averages on three independent samples: n=3). (**f**) ELISA Assay calibration curve for PSMA, performed with 1:2 serial dilution of the Reference Standard of the assay, reported as ng/ml in the x-axis. The linear interpolation fitting y = 0.0042 x + 0.0834 was used for the quantification of PSMA from the exosomal protein content. Quantification of PSMA by ELISA assay on exosomal protein extracts indicated a PSMA concentration of 7.85 µg/ml (total protein content: 1536 µg/ml), corresponding to 0.51% of the total protein content in weight. The experimental LOD for PSMA using a commercially available ELISA assay was 3.32 ng/mL, which corresponds to a molar concentration of 33.2 pM, in line with the producer specifications (28.1 pM).

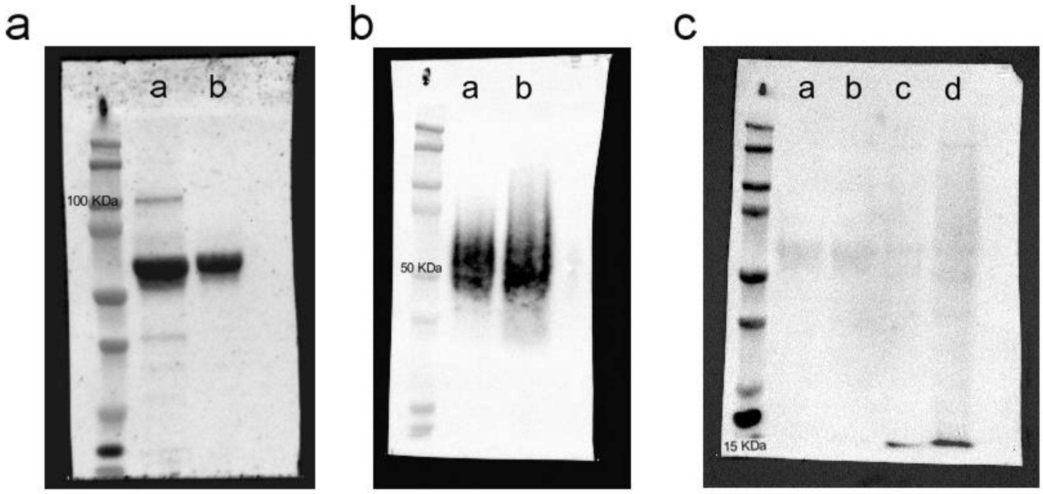

Figure S9 (**a**), (**b**), (**c**) are the uncut Western Blots for for the ones reported in S8 (**a**), S8 (**b**) and S8 (**c**), respectively; the lane most at left contains the weight scale from the Precision Plus Protein Dual Color Standards (Bio-Rad®) ladder. (**a**) PSMA blotting, at a molecular weight of 100 KDa, resulted positive for LNCaP SEV (lane a) and negative for PC-3 SEV (lane b); (**b**) CD63 blotting was positive both in LNCaP (lane a) and in PC-3 SEVs (lane b); (**c**) TOM20 blotting was negative in both LNCaP and PC-3 SEVs (lanes a and b) positive in the respective cell line (lane c d).

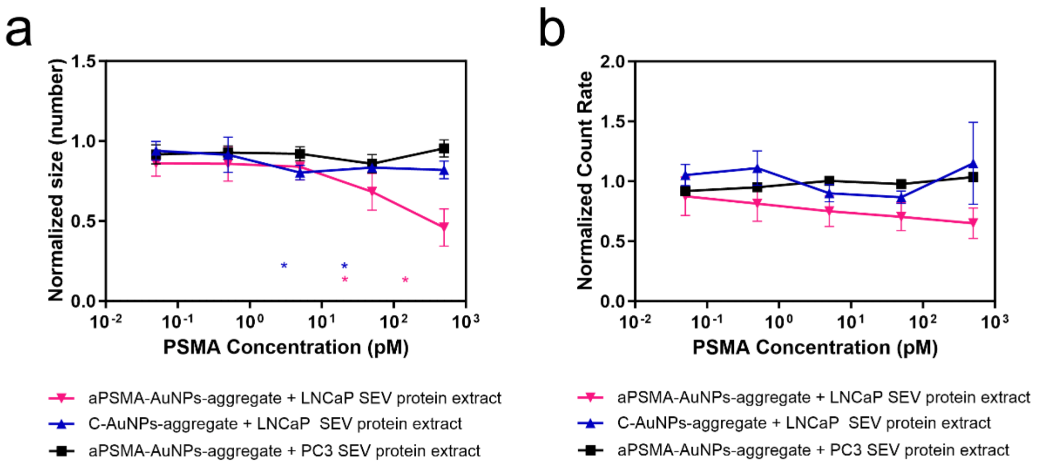

Figure S10 DLS recording of *in cuvette* response of the AuNPs-Aggregates when incubated with PSMA positive (LNCaP) or PSMA negative (PC3) small extracellular vesicles (SEVs) protein extracts. On the x-axis are reported equivalent PSMA concentrations calculated as if PSMA be 0.51% in weight of the total protein extract, also for PC3 SEVs protein extract (5 pM corresponds to 100 ng/ml of protein extract; its analysed concentrations ranged from 1 ng/ml to 10 µg/ml). When considering the normalized variation in size (**a**) we can observe a significant reduction in size in the two highest protein concentration values (50 and 500 pM of PSMA) for the aPSMA-AuNPs-Aggregate when incubated with LNCaP protein extract (red line and symbols), but also two significant points in the C-AuNPs-aggregate (blue line and symbols), even if in this case there is not a clear trend with protein concentration and the very small uncertainty on the second-last point is most probably fortuitous. On the other side, the Count Rate variation (**b**) does not follow the same trend of the size variation and does not show any significant different with respect to the initial condition. The presence of a protein extract dramatically increases the reaction mixture complexity in these samples and does not produce reliable DLS results for this experiment. P values (*, $p<0.05$) are for t-tests of -AuNPs-Aggregate incubated with SEVs extracts versus the case without SEVs extract). All measurements were repeated in triplicate (n=3) and error bars indicate the standard errors.

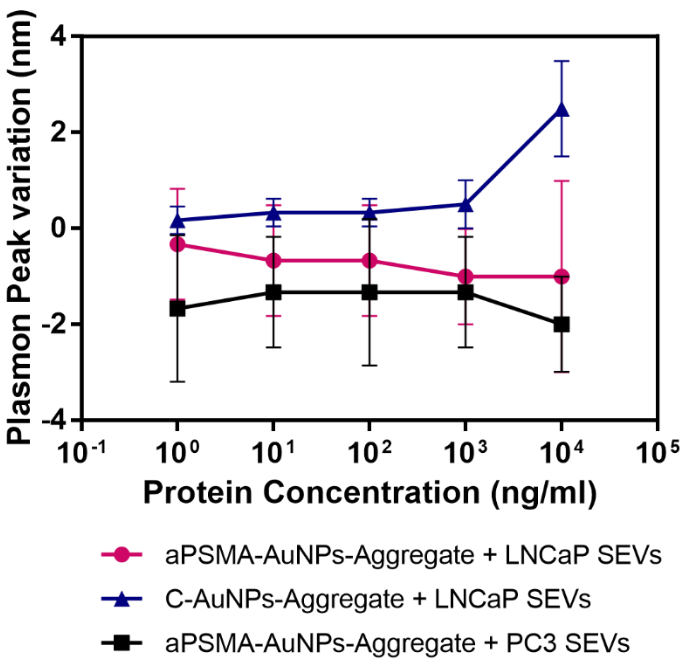

Figure S11 In cuvette response of the AuNPs-Aggregates when incubated with PSMA positive (LNCaP) or PSMA negative (PC-3) small extracellular vesicles (SEVs). No significant plasmon peak variation of the aPSMA-AuNPs-Aggregate was registered when the AuNPs-aggregates were incubated with intact PSMA positive extracellular vesicles. The total protein content in the analysed SEVs suspensions ranged from 1 ng/ml to 10 µg/ml, corresponding to a PSMA concentration range from 50 nM to 500 pM for LNCaP SEVs. All measurements were repeated in triplicate (n=3) and error bars indicate the standard errors.
